# Health Promoting Lifestyle and its Relationship with Self-Efficacy in Iranian Mastectomized Women

**DOI:** 10.31557/APJCP.2020.21.6.1667

**Published:** 2020-06

**Authors:** Monireh Hamed Bieyabanie, Mojgan Mirghafourvand

**Affiliations:** 1 *Midwifery Department, Tabriz University of Medical Sciences, Tabriz, Iran. *; 2 *Social Determinants of Health Research Center, Tabriz University of Medical Sciences, Tabriz, Iran.*

**Keywords:** Self-efficacy, mastectomy, breast cancer, health-promoting lifestyle

## Abstract

**Introduction:**

Lifestyle modification has an important role in long-term health of breast cancer patients. As a result, this study aimed to identify the health-promoting lifestyle and its subdomains in mastectomized women and its relationship with self-efficacy.

**Materials and Methods:**

This cross-sectional study investigated 100 mastectomized women in Tabriz-Iran, 2018. The participants were selected using the convenience sampling method. Data was collected using a sociodemographic questionnaire, the Health Promoting Lifestyle Profile II (HPLP-II), and the General Self-Efficacy Scale by Sherer. The multivariate general linear model with adjusting the sociodemographic variables was used to determine the relationship of the health-promoting lifestyle with self-efficacy.

**Results:**

The mean±SD total score of the health-promoting behaviors was 135.5±16.7 from the obtainable score of 52 to 208. The highest and lowest mean scores were observed in the spiritual growth (25.4±4.3) and physical activity (15.2±4.4), respectively. The mean±SD self-efficacy score in this study was 57.3±7.4 from the obtainable score of 17 to 85. There was a significant positive correlation between the total score of the health-promoting lifestyle (r= 0.369; p<0.001) with self-efficacy. Results from the adjusted general linear model showed that the age, educational attainment of the spouse, and self-efficacy were the health-promoting lifestyle predictors.

**Conclusion:**

The findings of this study indicate the importance of self-efficacy and modifiable variables such as education in the engagement of mastectomized women in the health-promoting lifestyle. Regarding the positive relationship of self-efficacy with the health-promoting lifestyle, it seems that the interventional attempts to improve self-efficacy in these patients especially with considering the variables of age and spouse’s educational level can contribute to the improvement of the health-promoting lifestyle.

## Introduction

Breast cancer is the most common malignancy and one of the leading cause of mortality in women (Ferlay et al., 2015). It is a multifactorial disease with different causes (Zendehdel et al., 2018). Although it is a global health issue, its prevalence, mortality rate, and survival rate considerably differ in different parts of the world, which can be due to many factors, such as population structure, lifestyle, genetic, and environmental factors (Hortobagyi et al., 2005).

Surgery is one of the most common treatments for breast cancer (Brunner, 2010). In total, mastectomy accounts for 81% of the breast cancer treatments in Iran (Najafi et al., 2005). It is a long-term, difficult, and frustrating process, which can cause physical, psychological, and social problem .The patient may face with problems in relation to self-care activities and healthy lifestyle (Guner and Kaymakci, 2014). Strong support, positive mental attitude, and encouragement of active living should be provided to women going through this process to help them restore balance in their life (Sagen et al., 2009). Despite increased survival rate, breast cancer survivors are at a greater risk of its recurrence and other serious diseases (Adams et al., 2015).The survivors’ lifestyle can affect their health and wellbeing. Physical exercise can reduce fatigue, and improve functional abilities, immunity, and quality of life which, in turn, is associated with improved survival rate and reduced risk of recurrence (Newton and Galvao, 2008). A healthier diet is associated with lower risk of recurrence, prolonged survival, and reduced risk of secondary cancers (Vrieling et al., 2013). The health-promoting lifestyle is a major criterion to determine the health status and is known as an underlying factor in preventing many diseases; in addition, the health promotion and disease prevention are directly affected by these behaviors (Baheiraei et al., 2012). Health-promoting behaviors are activities to empower people for increasing their control over self and society health (Mo and Winnie, 2010). 

Self-efficacy is a factor that causes general health promotion. This factor in patient with breast cancer can lead to better adaptation with the diagnosis and treatment process, improved mental imagination, and strengthened relationship between medical staff and patients (Liang et al., 2016). Today, it is believed that to achieve behavior change and higher health level, people should perceive themselves efficient in breaking barriers to behavior change (Baheiraei et al., 2013). 

A high sense of self-efficacy causes increased individual effort, higher [physical] resistance, and greater flexibility. As compared to people with low self-efficacy, those people with high self-efficacy feel more empowered. It is an important internal factor for preserving a long-term control over chronic diseases (Senecal et al., 2000). 

Based on our searches, there is no study about health promoting behaviors and the role played by self-efficacy in encouraging such behaviors among Iranian mastectomized women. To improve the lifestyle of this group and their health, it is necessary to obtain more knowledge on their health promoting lifestyle status as well as their self-efficacy as an effective factor to examine these behaviors. Therefore, since the incidence of breast cancer is growing in Iran (Rafiemanesh et al. 2016) and the control of complications and problems not only promotes survival rate but also builds a more cohesive family structure, this study aimed to determine the health-promoting lifestyle status and its relationship with self-efficacy in mastectomized women to maintain or even promote their health status. 

## Materials and Methods

*Study design and participants*

This descriptive-analytical cross-sectional study was conducted on 100 mastectomized women visiting the Breast Cancer Support Association and Shahid Ghazi Tabatabaei Hospital of Tabriz-Iran in 2018. 

The inclusion criteria were patients aged below 60 years, who underwent mastectomy between 1 and 5 years ago. The exclusion criteria were patients with self-reported history of acute mental disorder (depression, bipolar disorder, schizophrenia, etc.), taking psychiatric medications or other psychedelics, having other types of cancers and experiencing a recent tragic event.

The sample size was estimated based on the Guner and Kaymakci’s (2014) study according to the health-promoting lifestyle. In that, the sample size was estimated at 93 based on the highest standard deviation of subdomains (spiritual subdomain) with sd= 4.64, d=0.02, α=0.05, and m=47.0. To compensate for probable sample loss, the final sample size was considered 100. 

*Sampling*

This study was conducted after approving by the Ethics Committee of Tabriz University of Medical Sciences (ethics code: 1396900.TBZMED.REC). The sampling was done using the convenience sampling among the patients visiting the Breast Cancer Support Association and Shahid Ghazi Tabatabaei Hospital of Tabriz-Iran. To this end, the researcher attended at the sampling sites and selected the eligible mastectomized women using the convenience sampling method. The research objectives and methodology were explained to the participants and the informed written consent was obtained from the participants. Then, a sociodemographic characteristics questionnaire, the Health Promoting Lifestyle Profile-II (HPLP-II), and the General Self-Efficacy Scale of Sherer were completed by the researcher through interview by participants.

*Data collection tool*

This study used following questionnaires for data collection:

The sociodemographic characteristics questionnaire included items on age, marital status, number of children, time elapsed from the surgery, stage of the disease, educational attainment of the patient, job, educational attainment of the spouse, spouse job, Body Mass Index (BMI), income, place of living, and satisfaction of life. Validity of this questionnaire was measured using content and face validity.

The HPLP-II was developed by Walker et al., (1987) based on the Pender’s model to assess the health-promoting behaviors. This questionnaire is comprised of 52 items on six subscales of the health-promoting lifestyle, namely nutrition, physical activity, spiritual growth, health responsibility, stress management, and interpersonal relationships. All items are scored on a 4-point Likert scale anchored by 1”never,” 2”sometimes,” 3”often,” and 4”always.” The score of each subscale is obtained separately and then the total score of the questionnaire is calculated. This questionnaire includes nine items on nutrition, eight on physical activity, nine on spiritual growth, nine on health responsibility, eight on stress management, and nine on interpersonal relationships. This instrument has been translated into different languages with confirmed reliability and validity. The Farsi version of this instrument was used in a sample of 1359 women of reproductive age and its Cronbach’s alpha was obtained 0.9 (Mirghafourvand et al., 2014). In the present study, the reliability of the HPLP-II was confirmed by obtaining the internal cohesion (Cronbach’s alpha) equal with 0.84.

The self-efficacy was measured using the General Self-Efficacy Scale of Sherer. This scale has 17 items scored based on a Likert-scale from completely disagree to completely agree. In this scale, each item is scored from 1 to 5. Items 2, 4, 5, 6, 7, 10, 11, 12, 14, 15, 16, and 17 are scored inversely. As a result, the maximum and minimum scores are 85 and 17, respectively. This scale was developed by Barati (1997) and translated and validated in Iran with the Cronbach’s alpha of 0.79.

*Data analysis*

Collected data was analyzed using SPSS version 21. The descriptive statistics including frequency and percentage, mean, and standard deviation were used to describe sociodemographic information, health-promoting lifestyle, and self-efficacy. The Pearson correlation test was used to determine the relationship between health-promoting lifestyle and its subscales with self-efficacy. The one-way ANOVA and independent t-test were used to determine the relationship between sociodemographic characteristics and health-promoting lifestyle. The general linear model was used to determine the effect of each independent variable (sociodemographic characteristics and self-efficacy) on the dependent variables (total score of health-promoting lifestyle) and also to explain the variance.

## Results

The mean±SD of the age of the participants were 48.3±8.4 with 50% of them in the age group of 36-50. Other sociodemographic and obstetrics information of the participants are presented in Table 1.

The mean±SD of the total score of the health-promoting lifestyle was 135.5±16.5 from the obtainable score of 52 to 208. The highest and lowest mean scores were obtained in the spiritual growth (25.4±4.3) and physical activity (15.2±4.4), respectively. The mean±SD of the self-efficacy score in this study was 57.3±7.4 from the obtainable score of 17 to 85. The self-efficacy had positive correlation with the total score of the health-promoting lifestyle (r= 0.369; p<0.001) and its subdomains (r= 0.046 to 0.359; p<0.05); whereas, it was poorly correlated with physical activity (r=0.294), health responsibility (r=0.258), and stress management (r=0.283) and moderately correlated with spiritual growth (r= 0.359) (Table 2). 

Results from the one-way ANOVA and independent t-test showed a significant relationship of age, educational attainment of the patient and her spouse, and income with health-promoting lifestyle (p<0.05). Results from the adjusted general linear model showed that the age, educational attainment of the spouse, and self-efficacy were the predictors of the health-promoting lifestyle and explained 36.4% of variance in the total score of health-promoting lifestyle.

**Table 1:** The Relationship between Socio-Demographic Characteristics and Total Score of the Health-Promoting Lifestyle (n=100)

Variable	n (%)	mean±SD	P	Variable	n (%)	mean±SD	P
Body Mass Index (kg/m^2^)			0.926	Marital life duration			0.064
18.5 – 24	25 (25.0)	136.2±17.9		< 10	9 (9.0)	136.2±15.9	
25 – 29	43 (43.0)	134.7±15.3		10-20	11(11.0)	124.9±13.9	
≥ 30	32 (32.0)	135.9±18.1		> 20	79 (79.0)	137.3±16.5	
Education			0.004	Husband education			0.031
Elementary	32 (32.0)	127.8±14.6		Secondary and lower	24 (24.0)	130.7±16.8	
Secondary	14 (14.0)	138.9±13.1		High school	18 (18.0)	133.8±14.2	
High school	14 (14.0)	144.2±18.5		Diploma	30 (30.0)	134.4±15.6	
Diploma	22 (22.0)	133.3±15.8		University	26 (26.0)	143.7±17.1	
University	18 (18.0)	142.3±17.5		Time after surgery			0.648
Marital status				<2	15 (15.0)	137.2±16.9	
Single	11 (11.0)	138.1±17.3	0.593	4	36 (36.0)	133.5±15.7	
Married†	89 (89.0)	135.2±16.8		2-4	49 (49.0)	136.4±17.6	
Job			0.663	Disease stage			0.695
Housewife	88 (88.0)	133.9±16.2		Stage1	7 (7.0)	139.7±9.8	
Employed	12 (12.0)	147.3±16.7		Stage 2	88 (88.0)	135.4±17.2	
Husband job			0.264	Stage 3	5 (5.0)	131.4±16.9	
Working‡	29 (29.0)	139.1±15.6		Income level			0.004
Employee	18 (18.0)	129.7±14.7		Enough	22 (22.0)	141.7±19.3	
Shopkeeper	12 (12.0)	133.5±16.7		Not enough	28 (28.0)	127.1±13.3	
Other^☐^	40 (40.0)	136.9±17.5		Somewhat enough	50 (50.0)	137.4±15.8	
Life satisfaction			0.082	Child number			0.085
Totally satisfied	26 (26.0)	139.9±18.2		1	15 (15.0)	134.4±18.3	
Partially satisfied	58 (58.0)	135.9±15.8		2	50 (50.)	139.3±15.0	
No satisfied	15 (15.0)	127.8±15.4		3	34 (34.0)	131.3±17.1	
Age			0.016				
< 35	9 (9.0)	121.8±5.1					
36-50	49 (49.0)	134.7±17.3					
> 50	42 (42.0)	139.2±16.3					

*, Standard Deviation; †, Five participant was widowed; ‡, 7 people were unemployed; ^☐^, Including jobs such as salesman, driver, etc.

**Table 2:** The Status of Health-Promoting Lifestyle, its Subscales and Self-Efficacy and Their Relationships (n = 100)

Variable	Mean±SD*	Obtainable range	obtained practical range	Correlate with self-efficacy r (p)
Self-efficacy	57.3±7.4	17 to 85	37.0 to 74.0	-
HPLP total score	135.5±16.8	52 to 208	102.0 to 135.5	0.369 (0.001)
Nutrition	25.3±3.5	8 to 32	16.0 to 34.0	0.164 (0.156)
Physical activity	15.2±4.4	9 to 36	8.0 to 36.0	0.294 (0.010)
Interpersonal relationship	25.4±3.6	9 to 36	17.0 to 33.0	0.046 (0.690)
Stress management	19.9±3.3	8 to 32	14.0 to 30.0	0.283 (0.013)
Spiritual growth	25.4±4.3	9 to 36	12.0 to 35.0	0.359 (0.001)
Health responsibility	24.2±4.5	9 to 36	14.0 to 36.0	0.258 (0.012)

*, Standard Deviation

**Table 3:** Predictors of Health-Promoting Lifestyle Based on Multivariate General Linear Model (n= 100)

Variable	Β (95% CI*)	*P*-value
Self-efficacy	0.5 (0.1 to 0.1)	< 0.014
Age		
>50 (Reference)	0	
< 35	-17.9 (-30.2 to -5.6)	0.005
36-50	-5.0 (-12.0 to 1.9)	0.156
Husband’s education		
Academic (Reference)	0	
Elementary and secondary school	-1.4 (-12.6 to 9.2)	0.789
High school	1.4 (-9.1 to 12.0)	0.786
Diploma	-8.9 (-17.4 to -0.4)	0.039
Husband’s job		
Other (Reference)	0	
Working	6.3 (-1.3 to 14.0)	0.105
Employee	4.6 (-4.8 to 14.2)	0.328
Shopkeeper	-1.5 (-11.6 to 8.5)	0.758
Job		
Employee (Reference)	0	
Housewife	-8.4 (-10.3 to 5.5)	0.186
Women's education		
Academic (Reference)	0	
Elementary	-2.7 (-16.5 to 10.9)	0.687
Secondary school	6.9 (-7.2 to 21.1)	0.329
High school	13.9 (-1.3 to 29.2)	0.072
Diploma	4.2 (-7.8 to 16.3)	0.489

*, 95% Confidence Interval

## Discussion

The results of this study showed that the mean score of the health-promoting lifestyle was at a moderate level. The highest and lowest mean scores of the participants were obtained in the spiritual growth and physical activity, respectively. The educational attainment of the spouse, self-efficacy, and age were determined as predictors of the health-promoting lifestyle.

In this study, the mean score of the health-promoting lifestyle was at the moderate level, which is consistent with the studies by Yi and Kim (2013) on breast cancer survivals in Seoul, Baheiraei et al., (2014) on women at childbearing age in Tehran (capital of Iran), and Mehri et al., (2016) on students in Sabzevar-Iran. The risk of future health complications in patients receiving cancer treatment may be due to genetic or lifestyle factors. The behavior change is not typically maintained after cancer diagnosis. As a result, the unhealthy behavioral habits and undesirable consequences in cancer survivors can be managed by the third prevention (Demark-Wahnefried et al., 2006; Demark-Wahnefried et al., 2008).

The highest and lowest mean scores in this study were obtained, respectively, for the spiritual growth and physical activity. Consistent with the current study, a study on the middle-aged Iranian women (Enjezab et al., 2012) and breast cancer survivors (Demark-Wahnefried et alk., 2006) showed that the highest and lowest mean scores were for the spiritual growth and physical activity. The prospective observational studies have shown that physical activity after cancer diagnosis is associated with reduced risk of recurrence and lower total mortality rate because of breast, colorectal, prostate, and ovarian cancers (Kenfield et al., 2011; Holmes et al., 2005; Haydon et al., 2006). After the completion of the cancer treatment process, it is essential to set lifetime goals for weight control, physically active lifestyle, and healthy diet to promote general health, quality of life, and lifespan (Coward et al., 2006). In general, results suggest that appropriate measures and strategies should be adopted to modify lifestyle of all women, specifically those with a cancer. As a result, in addition to pharmaceutical therapies, behavior and lifestyle modifications can be effective in promoting the treatment process in this group of women.

The self-efficacy is regarded as an effective factor of the health-promoting behaviors. People with strong self-efficacy are able to have a greater control over their physical and situational problems (Keedy et al., 2014). The results of Jackson et al.,’s study (2007) showed that health self-efficacy predicted the level of engagement of students in a health promoting lifestyle. A study on the health-promoting behaviors in patients with chronic renal diseases in Thailand showed a positive relationship between the perception of self-efficacy and the health-promoting behaviors (Polsingchan, 2010). Studies have shown a relationship between self-efficacy with health behaviors, such as dietary habits (Fisher and Kridli, 2014; Chang et al., 2008; Wall et al., 2012). In addition, a significant positive correlation has been shown between physical activity and self-efficacy (Geboers et al., 2014; Andenæs et al., 2014). As a result, regarding the role of self-efficacy in the promotion of the health-promoting lifestyle factors, it is needed to pay particular attention to self-efficacy improvement with the aim of health promotion of mastectomized women.

This study found age as a predictor of the health-promoting lifestyle in the mastectomized women, that women with the mean age of lower than 35 years had unhealthier lifestyle than women with the mean age over 50. This finding was consistent with the findings of Mirghaforvand et al., (2014) and Alcandri et al., (2008), that the age is a factor related to the health-promoting behaviors. Previous studies suggest that the health level improves with aging (Hulme et al., 2003; Huang et al., 2010; Hong et al., 2007). The improvement of the health-promoting behaviors with aging indicates the effect of life experience (Merluzzi et al., 2001).

In this study, educational attainment of the spouse was found to be a predictor of the health-promoting lifestyle. Women whose spouse had academic education reported healthier lifestyle. A study in the USA showed a relationship between educational attainment and health behaviors (Huang et al., 2010). Katapodi et al., (2005) and Drageset et al., (2002) showed that higher educational attainment causes stronger social support among the patients with breast cancer. It is probable that educational attainment can improve health behaviors through raising awareness of the treatment and health-promoting strategies.

*Limitation*

Among the research limitations was its cross-sectional nature, in that the relationships of self-efficacy and some sociodemographic characteristics with the health-promoting lifestyle does not necessarily indicate a causal relationship. Another limitation was the use of convenience sampling method that reduced the generalizability of the results. Among the research strengths was the use of a standard questionnaires to assess the health-promoting lifestyle and self-efficacy. Regarding the importance of health-promoting behaviors in cancer patients, it is recommended to conduct similar studies in patient with other types of cancers, and design and implement appropriate solutions to improve health-promoting behaviors based on the results of the current study and the literature.

In conclusion, the findings of this study indicate the importance of self-efficacy and modifiable variables such as education in the engagement of mastectomized women in the health-promoting lifestyle. Regarding the positive relationship of self-efficacy with the health-promoting lifestyle in mastectomized women, the counseling, educational, and interventional programs should be designed to enhance self-efficacy and implement the health-promoting lifestyle behaviors in hospitals and healthcare centers.
